# Improved Geometry of Decellularized Tissue Engineered Heart Valves to Prevent Leaflet Retraction

**DOI:** 10.1007/s10439-015-1386-4

**Published:** 2015-07-17

**Authors:** Bart Sanders, Sandra Loerakker, Emanuela S. Fioretta, Dave J.P. Bax, Anita Driessen-Mol, Simon P. Hoerstrup, Frank P. T. Baaijens

**Affiliations:** Department of Biomedical Engineering, Eindhoven University of Technology, Postbus 513, 5600 MB Eindhoven, The Netherlands; Institute for Complex Molecular Systems, Eindhoven University of Technology, Eindhoven, The Netherlands; Equipment & Prototype Center, Eindhoven University of Technology, Eindhoven, The Netherlands; Swiss Center for Regenerative Medicine, University Hospital of Zürich, Zurich, Switzerland

**Keywords:** Heart valve replacement, Tissue engineering, Computational modeling, Decellularization, Bioreactor

## Abstract

Recent studies on decellularized tissue engineered heart valves (DTEHVs) showed rapid host cell repopulation and increased valvular insufficiency developing over time, associated with leaflet shortening. A possible explanation for this result was found using computational simulations, which revealed radial leaflet compression in the original valvular geometry when subjected to physiological pressure conditions. Therefore, an improved geometry was suggested to enable radial leaflet extension to counteract for host cell mediated retraction. In this study, we propose a solution to impose this new geometry by using a constraining bioreactor insert during culture. Human cell based DTEHVs (*n* = 5) were produced as such, resulting in an enlarged coaptation area and profound belly curvature. Extracellular matrix was homogeneously distributed, with circumferential collagen alignment in the coaptation region and global tissue anisotropy. Based on *in vitro* functionality experiments, these DTEHVs showed competent hydrodynamic functionality under physiological pulmonary conditions and were fatigue resistant, with stable functionality up to 16 weeks *in vivo* simulation. Based on implemented mechanical data, our computational models revealed a considerable decrease in radial tissue compression with the obtained geometrical adjustments. Therefore, these improved DTEHV are expected to be less prone to host cell mediated leaflet retraction and will remain competent after implantation.

## Introduction

Annually, approximately 280.000 patients worldwide undergo either a mechanical- or bio-prosthetic heart valve transplantation.^20^ Although these are life saving devices, the lack of growth potential of these prostheses is a major problem for pediatric patients. They have to go through multiple staged interventions to accommodate the increased annulus size, with increasing morbidity and mortality risks.^22^ Therefore, there is an urgent need for heart valve prostheses with growth capacity that last a lifetime.^3^,^21^

Decellularized tissue-engineered heart valves (DTEHVs) might represent a promising alternative. From our first long-term *in vivo* experiments, where we implanted DTEHVs in sheep and non-human primates, we learned that the DTEHVs start to repopulate after 5 h, accompanied by changes in the extracellular matrix after 8 weeks of implantation. Moreover, there was ECM production over time, indicative for tissue regeneration and growth potential.^6^,^30^ This in contrast to decellularized xenogeneic heart valves, which only show limited host cell repopulation.^7^,^13^ Besides, these DTEHVs could be available off-the-shelf.^5^ Although these results are promising, there were signs of leaflet shortening and fusion of the leaflets with the wall, ultimately resulting in valvular insufficiency, an effect which is also reported by other groups.^8^,^10^ An explanation for this leaflet-fusing and shortening problem might be found in the valve geometry. It was shown from computational simulations by Loerakker *et al.*^15^ that the leaflets in this original valve design were subjected to tissue compression in radial direction when loaded under physiological pulmonary pressures. It might explain why this particular valve geometry, in combination with infiltrated host cell induced remodeling, eventually resulted into reduced leaflet size. Based on these findings, Loerakker *et al.* also suggested an improved valve geometry that should enable radial leaflet extension during hemodynamic loading to counteract for cellular retraction forces. This required a more profound belly curvature, enhanced coaptation area and predominantly circumferential collagen orientation.^15^ However, controlling the geometry of tissue engineered heart valves (TEHVs) during culture was limited thus far. Regardless of the initial shape of the scaffold starter matrix, tissue compaction occurred in all possible unconstrained directions in response to the traction forces exerted by the vascular derived cells (myofibroblasts) used to culture the valves.^29^ This resulted in a flattened leaflet configuration, and absence of coaptation area after culture.^12^,^17^

Therefore, the aim of this study is to find a solution to be able to improve, impose and maintain the DTEHV geometry, in accordance with the suggested geometry from the computational simulations, to reduce leaflet tissue compression in radial direction under pulmonary loading conditions. A bioreactor insert matching the improved geometry was developed, which will function as an overall geometric constraint during culture. In this way, the leaflets will compact themselves around the bioreactor insert, and when removing the insert after the decellularization procedure, the DTEHV is likely to maintain its shape. This makes it possible to design, impose and maintain the desired DTEHV geometry. Human cell-based DTEHVs were produced, and their functionality and stability were assessed using hydrodynamic and fatigue *in vitro* tests. The effects of the bioreactor insert on tissue formation and collagen orientation were investigated using histology and confocal microscopy. Furthermore, the mechanical properties were analyzed to investigate the degree of tissue anisotropy and used as input for computational simulations on leaflet tissue loading behavior, in order to analyze the radial strain distribution in the newly designed DTEHV.

## Materials and Methods

### Insert Manufacturing and Positioning

Based on the mathematical description of Hamid *et al.*^11^ the original valve design was improved by adding coaptation and increasing the curvature in the belly region as being previously described by Loerakker *et al.*^15^ This improved geometry was exported as a STEP file from the simulation software (Abaqus 6.10 Simulia, USA) and imported into computer-aided design software (Autodesk Inventor, USA), to make a one-piece component of 27.8 mm in length and 29.7 mm in diameter, which was compatible with the diastolic pulse duplicator (DPD) bioreactor system.^17^ The bioreactor inserts were made out of a solid piece of polyether ether ketone (PEEK) by using computer controlled milling technology.

The insert was positioned at the arterial side of the valve to enable tissue compaction around the individual posts. Small holes (0.5 mm in diameter and 1 mm spacing) are covering the insert wall to facilitate nutrient exchange with the adjacent tissue. Three large triangular shaped openings on top were incorporated for medium exchange towards the tissue-engineered valvular wall.

### Heart Valve Tissue Engineering

Tissue-engineered heart valves (TEHVs) (*n* = 5) were cultured as previously described.^17^ In short, tri-leaflet heart valves were cut from non-woven polyglycolic-acid meshes (PGA; thickness 1.0 mm; specific gravity 70 mg/cm^3^; Cellon, Luxembourg), sewn (Prolene 6-0, Ethicon, USA) into a radially self expandable nitinol stent (length = 31 mm, ID = 30 mm at 37 °C; PFM-AG, Germany), and coated with 1% poly-4-hydroxybutyrate (P4HB; MW: 1 × 10^6^; TEPHA Inc., USA) in tetrahydrofuran (THF; Sigma-Aldrich, USA). The heart valve shaped constructs had an initial coaptation length of 5 mm and a maximal radial belly length of 19 mm. These constructs were sterilized with 70% ethanol (EtOH, VWR international S.A.S. Fontenay-Sous-Bois, France) for 15 min, washed 3 times with phosphate buffered saline (PBS) for 10 min, incubated in an antibacterial-anti fungus solution 10% penicillin/streptomycin (Pen/Strep) (Lonza, Belgium), with 0.5% fungin (InvivoGen, USA), for 30 min, and washed 3 times with PBS for 10 min. Hereafter, the valves were incubated overnight in growth medium (Advanced Dulbecco’s Modified Eagle Medium, Gibco, USA), supplemented with 10% fetal bovine serum (FBS, Biochrom, Germany), 1% Pen/Strep and 1% Glutamax (Gibco, USA). Primary isolated human vascular-derived cells were harvested from the human vena saphena magna from a 77-year-old patient, according to the Dutch guidelines for secondary used materials, and seeded (0.3 × 10^6^ cells/cm^2^, passage 6) into the valvular shaped scaffolds using fibrin as a cell carrier. The seeded constructs were placed into the DPD bioreactor system together with the newly developed insert, containing growth medium supplemented with L-ascorbic acid 2-phosphate (0.25 mg/mL, Sigma, USA). Pulsatile flow was applied at 1 Hz to the unshielded ventricular side of the valve.

### Decellularization Procedure

After 4 weeks of culture, the obtained TEHVs (*n* = 5) were decellularized as described by Dijkman *et al.*^5^ Briefly, TEHVs were washed 3 times 10 min with PBS and decellularized overnight in detergent solution (0.25% Triton X-100, sodium deoxycholate and 0.02% EDTA), where after the bioreactor insert was removed. Nucleic remnants were enzymatically degraded by using Benzonase (EMD Millipore, USA) incubation steps, diluted in 50 mM TRIS–HCL buffer solution in concentrations of 100, 80 and 20 U/mL for 8, 16 and 8 h, respectively, on a shaker at 37 °C. Afterwards, the DTEHVs were washed 3 times with PBS and incubated for 24 h in M-199 medium (Gibco, USA) on a shaker at 4 °C to remove cellular remnants. Valves were washed 3 times with PBS, sterilized with 70% EtOH for 15 min, washed 3 times with PBS, and incubated for 30 min with an anti-fungi/bacterial solution. After sterilization, the DTEHVs were stored at 4 °C until further use.

### *In-Vitro* Valve Functionality

Out of the 5 DTEHVs, 4 were used for *in vitro* valve functionality assessments, and the remaining valve served as a control, not subjected to fatigue testing.

#### Hydrodynamic Pulsatile Functionality Assessment

DTEHVs (*n* = 4) were placed inside a silicon annulus of 30 mm inner diameter and positioned into a hydrodynamic pulsatile test system (HDT-500, BDC Laboratories, USA) containing a physiologic saline solution at 37 °C. Valves were subjected to physiological pulmonary conditions (rate of 72 BPM, stroke of 70 mL, maximum diastolic pressure difference of 25 mmHg) for 1 h. Flow and pressures were measured using a transonic sensor (TS410, Transonic Systems, USA) and pressure sensors (BDC-PT, BDC Laboratories, USA), respectively. Data was collected for 3 s at 5 kHz, and functionality was assessed from an average over 10 cardiac cycles by using Statys™ software (BDC Laboratories, USA), to determine the regurgitation fraction, the effective orifice area (EOA), and cardiac output (CO). The regurgitation fraction was defined as being the sum of the leakage volume and the closing volume, expressed as a percentage of the stroke volume. Slow-motion movies were recorded at high-speed burst mode to assess opening and closing behavior of the valves in motion (G15 PowerShot, Canon, USA).

#### Fatigue Assessment

DTEHVs (*n* = 4) were placed inside a 30 mm diameter silicon annulus and positioned into a valve durability tester (VDT-3600i, BDC Laboratories, USA) containing physiologic saline solution at 37 °C. Valves were subjected to accelerated opening and closing cycles at a frequency of 10 Hz and a stroke between 1.20 and 2.10 mL. Proper opening and closing behavior was assessed by analyzing slow-motion recordings (G15 PowerShot, Canon, USA). During closure, the maximum differential loading was targeted at 28 mmHg and was automatically maintained. After each 3 × 10^6^ cycles, the DTEHVs were tested again for their hydrodynamic functionality by using the hydrodynamic pulsatile test system as described above.

### Qualitative Tissue Analysis and Global Collagen Orientation

#### Histology

Middle sections of the valves (*n* = 5) were fixed overnight in 3.7% formalin (Fluka, USA) at 4 °C. After washing in PBS, the samples were embedded in Tissue-Tek (Sakura, the Netherlands) and cured gradually in liquid nitrogen vapor. Cryosections were cut at 10 μm thickness and stained for Hematoxylin and Eosin (H&E) to assess general tissue formation and the effectiveness of the decellularization procedure, Masson Trichrome (MTC) (Staining kit, Sigma, USA) for the presence and distribution of collagen, and Elastica van Gieson (EvG) (Staining kit, Merck, Germany) for the presence of elastic matrix formation. The samples were embedded in Entellan (Merck, Germany) and analyzed using bright field microscopy (Axio Observer Z1, Zeiss, Germany) in the mid regions of the heart valve leaflets.

#### Confocal Microscopy

Half leaflet sections of all (*n* = 5) valves were analyzed for the effect of the insert on the global collagen orientation. Samples were stained using a whole mount staining with the collagen specific dye CNA35-OG488,^14^ for 1 h in PBS and visualized with a confocal microscope (TCS SP5X, Leica, Germany). The dye has an excitation and emission profile of 488 and 500–550 nm, respectively. Samples immerged in Mowiol (Sigma, USA) were mounted between two preparation glasses. The specimen was observed with a ×10 objective and Z-stacks were made throughout 200 μm of the entire arterial side of the sample using stitched adjacent tile scans. Afterwards, a maximum intensity projection was made in *Z*-direction.

### Tissue Mechanics and *In Vivo* Collagen Remodeling Simulations

#### Biomechanical Analyzes

Mechanical properties of the control valve were analyzed by using a biaxial tensile tester (BioTester, 5 N load cell; CellScale, Waterloo, Canada) in combination with LabJoy software (V8.01, CellScale). Two square samples (36 mm^2^ each) per valve were symmetrically cut from the belly region. Sample thickness was measured at 3 random locations using an electronic caliper (CD-15CPX, Mitutoyo, Japan) and averaged. The samples were stretched equibiaxially in both the radial and circumferential direction up to 20% strain, at a strain rate of 100% per minute. After stretching, the samples recovered to 0% strain at a strain rate of 100% per minute, followed by a rest cycle of 54 s. Prior to measuring the final stresses, samples were preconditioned with 5 of these cycles. A high-order polynomial curve was fitted through each individual data set in both the radial and circumferential direction. The stiffness of the tissue was represented by the tangent modulus and was defined as the tangent to the fitted polynomial curve at 20% strain.

#### Computational Simulations

Based on the obtained experimental mechanical data of these improved DTEHVs, computational simulations (Abaqus 6.10 Simulia, USA) as described by Loerakker *et al.*^15^ were executed to examine if the implemented changes in valve geometry resulted in reduced radial compression under pulmonary pressure conditions. The original valve design was based on the mathematical description of Thubrikar^27^ with the parameters *R*_b_ = *R*_c_ = 13.5 mm, *H* = 19.15 mm, *H*_s_ = 3.15 mm and *β* = 0° without any coaptation. The improved valve geometry was described by Hamid *et al.*^11^ with the parameters *a* = *b* = 3.1 mm, *H* = 18 mm, *R* = 13.5 mm, with 5 mm coaptation. All values were obtained as described in the original paper^15^ and both designs used the experimentally measured tissue thickness of 0.58 mm.

The total stress in the tissue was given by the stress in the collagen fibers (with volume fraction *ϕ*_f_) and the isotropic matrix components (with volume fraction $\left( {1 - \phi_{\text{f}} } \right)$)^15^:

1 $$\varvec{\sigma}=\varvec{\sigma}_{\text{m}} +\varvec{\sigma}_{\text{f}}$$

The isotropic matrix stress is equal to:

2 $$\varvec{\sigma}_{\text{m}} = \left( {1 - \phi_{\text{f}} } \right)\left( {\kappa \frac{\ln J}{J}{\mathbf{I}} + \frac{G}{J}({\mathbf{B}} - J^{\frac{2}{3}} {\mathbf{I}}} \right)$$

with **B** = **F** · **F**^T^ the left Cauchy-Green tensor, **F** the deformation gradient tensor, *J* = det (**F**) the volumetric change ratio. The tissue was modeled as almost incompressible (υ = 0.498), and the shear modulus G was set to 10 kPa to prevent numerical instabilities. The compression modulus is defined as:

3 $$\kappa = \frac{{2G\;\left( {1 + \upsilon } \right)}}{{3\;\left( {1 - 2\upsilon } \right)}} .$$

The collagen fibers were modeled with an angular distribution of fibers (resolution of 3°), where the stress if given by:

4 $$\varvec{\sigma}_{\text{f}} = \sum\nolimits_{i = 1}^{N} {\varphi_{\text{f}}^{i} \sigma_{\text{f}}^{i} {\vec{\text{e}}}_{\text{f}}^{i} {\vec{\text{e}}}_{\text{f}}^{i} }$$

with N the number of fiber directions, *φ*_f_^*i*^ the collagen volume fraction in direction $i\left( {\sum\nolimits_{i = 1}^{N} {\varphi_{\text{f}}^{i} = \phi_{\text{f}} } } \right), \sigma_{\text{f}}^{i}$ the magnitude of the stress in each fiber direction, and ${\vec{\text{e}}}_{\text{f}}^{i}$ a unit vector in the deformed fiber direction *i*. The magnitude of the stress is a function of the fiber stretch *λ*_f_:

5 $$\sigma_{\text{f}} = \left\{ {\begin{array}{*{20}l} {k_{1} \lambda_{\text{f}}^{2} \left( {{\text{e}}^{{k_{2} \left( {\lambda_{\text{f}}^{2} - 1} \right)}} - 1} \right)} \hfill & {\text{for}} \hfill & {\lambda_{\text{f}} \ge 1 } \hfill \\ 0 \hfill & {\text{for}} \hfill & {\lambda_{\text{f}} < 1} \hfill \\ \end{array} } \right.$$

The collagen volume fractions are described with a Gaussian distribution:

6 $$\varphi_{\text{f}}^{i} = {\text{A}}\exp \left( {\frac{{ - \left( {\gamma^{i} - \mu } \right)^{2} }}{{2\sigma^{2} }}} \right)$$

with *γ*^*i*^ the angle of fiber direction *i* with respect to the circumferential direction, *μ* the main fiber angle (0°), *σ* the standard deviation, and A a scaling factor to ensure that $\sum\nolimits_{i = 1}^{N} {\varphi_{\text{f}}^{i}} = {\varphi_{\text{f}}}$. Parameters $k_{1} ,k_{2} ,\;{\text{and}}\;\sigma$ were fitted to the equibiaxial tensile test data of the control valve according to the least squares method. The effect of collagen anisotropy on tissue loading was investigated by comparing the experimentally determined collagen anisotropy to complete collagen isotropy. Eventually, the computational model simulated the radial and circumferential strains in the DTEHV leaflets, by applying a mean pulmonary differential pressure of 15 mmHg over the valve, using both the original as well as the improved geometry.

### Statistics

To analyze DTEHV fatigue behavior over time, a linear regression analysis was performed (Prism V6.0d, Graphpad, USA) on the closing volume, leakage volume, cardiac output, and the effective orifice area, with a slope being significantly non-zero for *p* < 0.05. Time points were averaged, and represented by their mean ± standard deviation.

For the biomechanical analysis, samples were averaged per valve, and represented by their mean ± standard deviation. The presence of anisotropy was defined as a significant difference (*p* < 0.05) between the obtained stiffnesses in both radial and circumferential direction, analyzed using a paired student *t* test (Prism V6.0d, Graphpad, USA).

## Results

### Production and Functionality of the Bioreactor Insert

#### Production of the Insert

**Figure 1:**
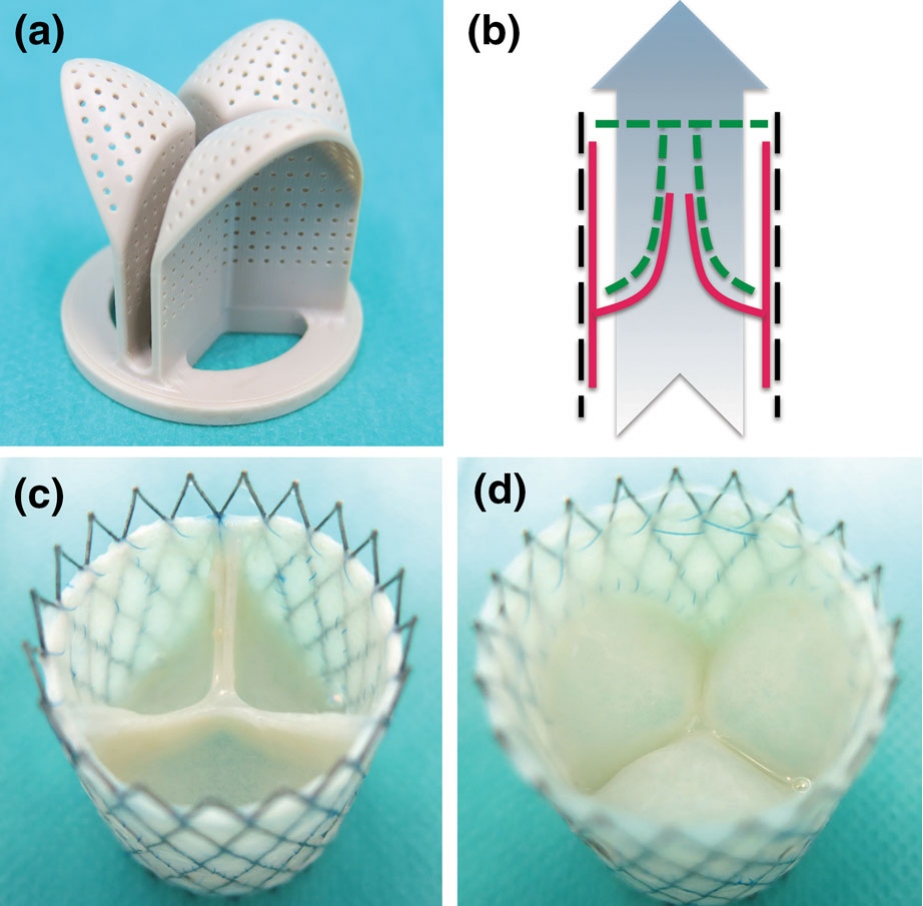
Bioreactor insert and representative DTEHV results. The developed bioreactor insert, used to impose and control the heart valve geometry during culture (a). A schematic representation of the positioning of the insert depicted in green, inside the bioreactor setup with the heart valve indicated in red, the nitinol stent in black and the pulsatile medium flow as a blue arrow (b). Two representative pictures of a human cell based DTEHVs cultivated by using the insert, from a top (c) and bottom view (d), showing large coaptation areas and an profound belly curvature, matching the shape of the bioreactor insert.

The improved heart valve geometry as suggested by the computational simulations, was successfully translated into a physical rigid object (Fig. 1a), matching the exact same geometry. The surface of the insert was smooth and the holes were equally distributed over the entire surface to enable sufficient nutrient exchange to the scaffold. There was sufficient space between the individual posts to prevent leaflet fusion during culture. The insert fitted nicely into the DPD bioreactor system without obstructing the pulsating flow passing by the ventricular side towards the arterial side (Fig. 1b).

#### Functioning of the Insert

During the 4 weeks of culturing, the leaflets of the TEHVs compacted tightly around the insert, adapting to the imposed geometry. After decellularization, the valves maintained their shape, and there was no leaflet retraction visible upon removal of the insert. The coaptation area was approximately 5 mm in length (Fig. 1c), and the belly region of the DTEHVs maintained the imposed curvature (Fig. 1d). Tissue was uniformly distributed throughout the leaflets, without any damage or other macroscopically detectable irregularities.

### Hemodynamic Functionality and Fatigue Resistance

**Figure 2:**
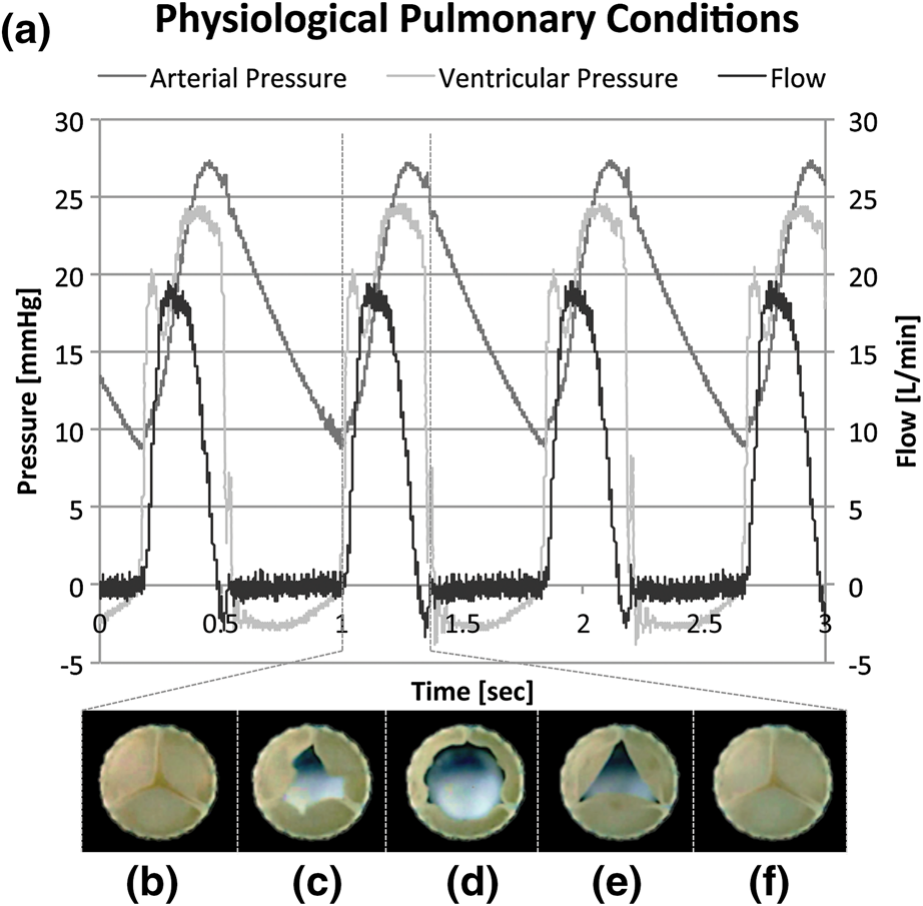
Physiological hydrodynamic pulmonary * in vitro* test results. Representative results of a DTEHV subjected to * in vitro* hydrodynamic physiological pulmonary pulsatile stimulation after 12 million cycles, showed competent valve functioning as indicated in the graph (a). Valves were opening completely (b–d) and closed symmetrically (e, f).

DTEHVs (*n* = 4) were subjected to physiological pulmonary conditions in a hydrodynamic test setup. A representative graph of one valve is shown in Fig. 2. Overall, all valves opened up completely (Figs. 2b–2d), and closed symmetrically (Figs. 2e–2f). Prolapse was not observed in any of the valves.

**Figure 3:**
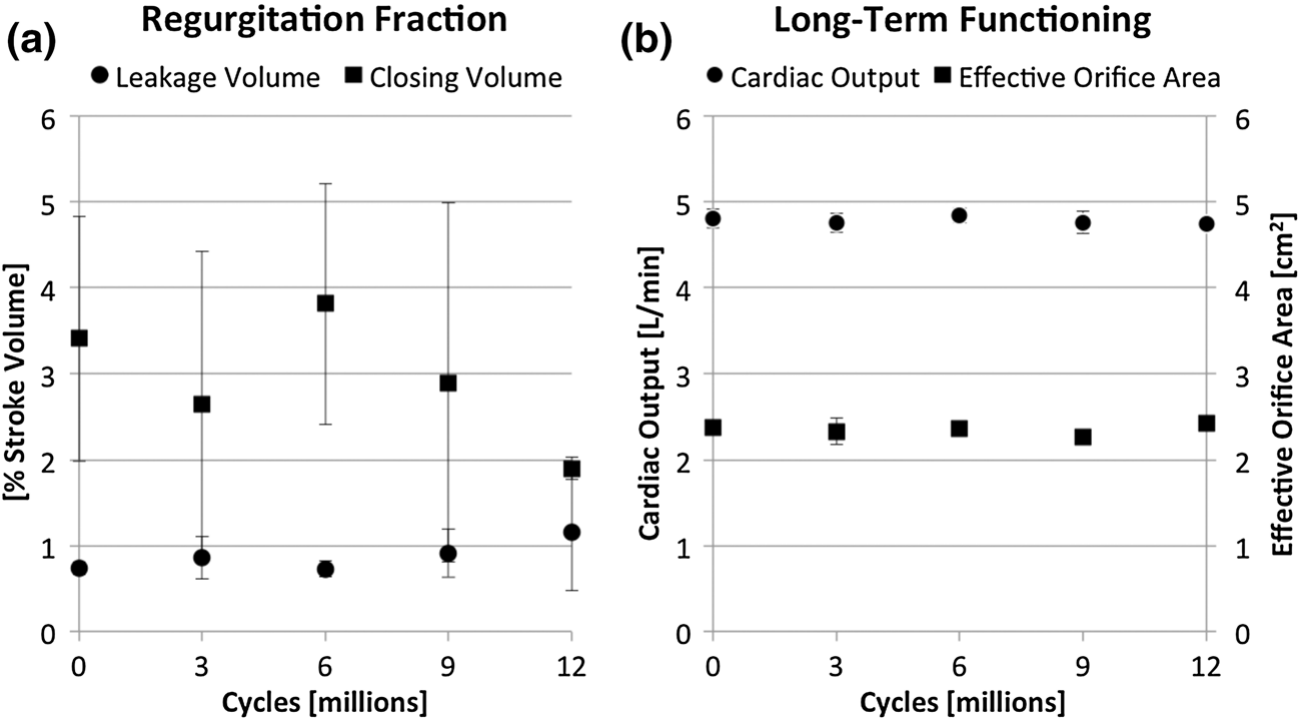
Long-term functional behavior of DTEHVs * in vitro*. Interval measurements of the regurgitation fraction, cardiac output, and effective orifice area, from start (*n* = 4), after 3 million cycles (*n* = 4), 6 million cycles (*n* = 3), 9 million cycles (*n* = 2), and after 12 million cycles (*n* = 2), showing no significant increase in regurgitation over time (*p* < 0.05) (a). Also the cardiac output and effective orifice area maintained stable over time (b). Values are represented as mean values ± standard deviation.

For the long-term durability assay, valve opening and closing behavior was set to be comparable to the physiological behavior as observed in the hydrodynamic setup. From the DTEHVs (*n* = 4) subjected to fatigue tests, three remained functional up to about 12 million cycles, representative for 16 weeks *in vivo* follow-up time. One valve failed at 4 million cycles, and was unable to maintain stable pressure conditions during fatigue testing from the start. The valves showed no decrease in functionality over time by having an initial regurgitation fraction of 4.13 ± 1.44%, consisting out of a closing volume percentage of 3.41 ± 1.42%, and a leakage volume percentage of only 0.73 ± 0.08% (Fig. 3a). The valves maintained a consistent effective orifice area of 2.37 ± 0.04 cm^2^, with a maintained cardiac output of 4.80 ± 0.11 L/min over time (Fig. 3b). Loss in functionality was sudden and in all cases the result of leaflet rupture at one of the commissure points.

### Qualitative Tissue Analysis and Global Collagen Orientation

#### Histological Appearance

**Figure 4:**
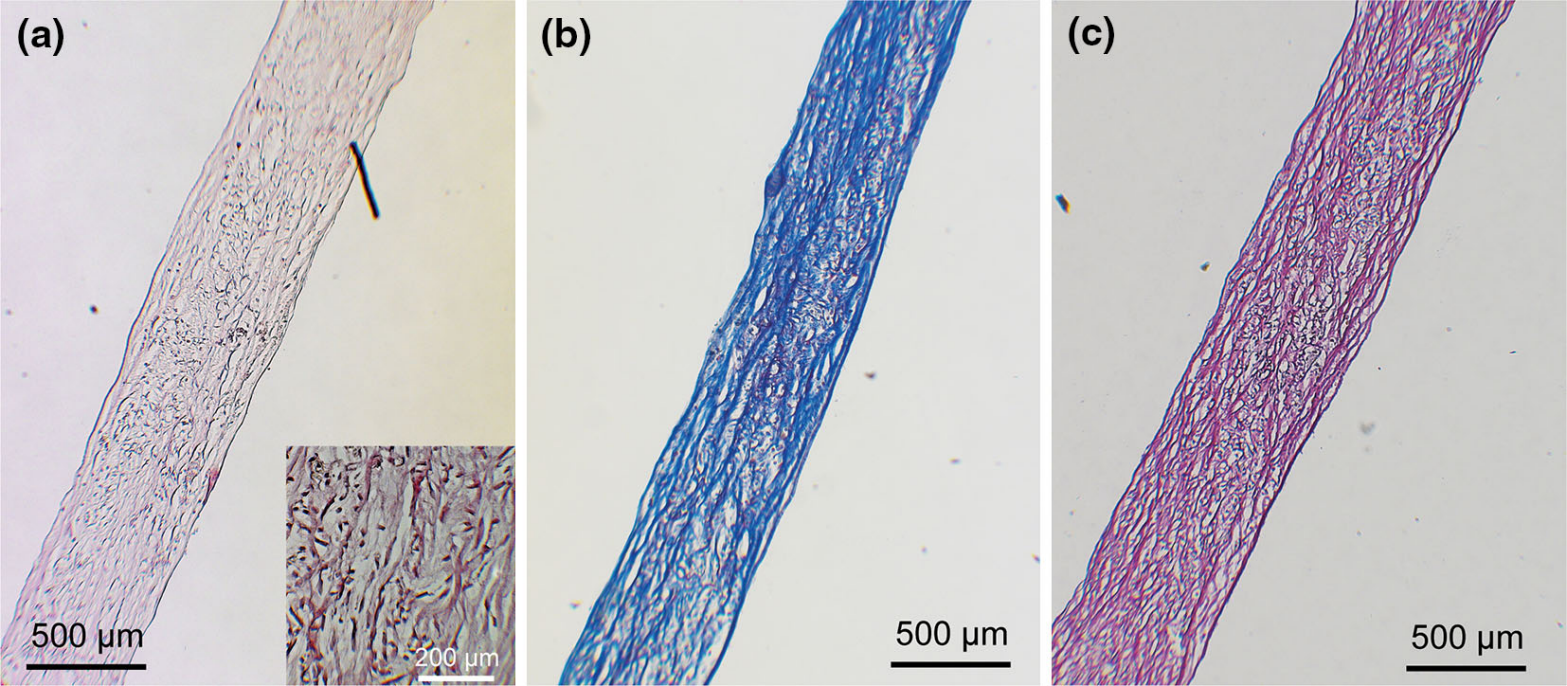
Histology on DTEHVs. Representative images of stained sections of DTEHV leaflets with Hematoxylin and Eosin, showing appropriate decellularization, equal tissue formation and the presence of scaffold remnants (a). Masson Trichrome revealed mainly collagen deposition depicted in blue (b), where Elastica van Gieson indicated no elastic matrix formation within the construct (c).

Histology was performed on samples of all DTEHVs. Representative photographs in Fig. 4, show that the tissue was uniformly distributed throughout the thickness of the leaflets. Furthermore, no necrotic or damaged tissue was observed. The decellularization procedure successfully removed all cells as demonstrated by the H&E staining, although scaffold remnants were still present in the tissues (Fig. 4a). From the MTC staining it appeared that the leaflets are mainly composed of collagen (Fig. 4b), with no signs of elastic matrix formation in the VvG staining (Fig. 4c), as would otherwise be indicated by black fibers.

#### Global Collagen Orientation

**Figure 5:**
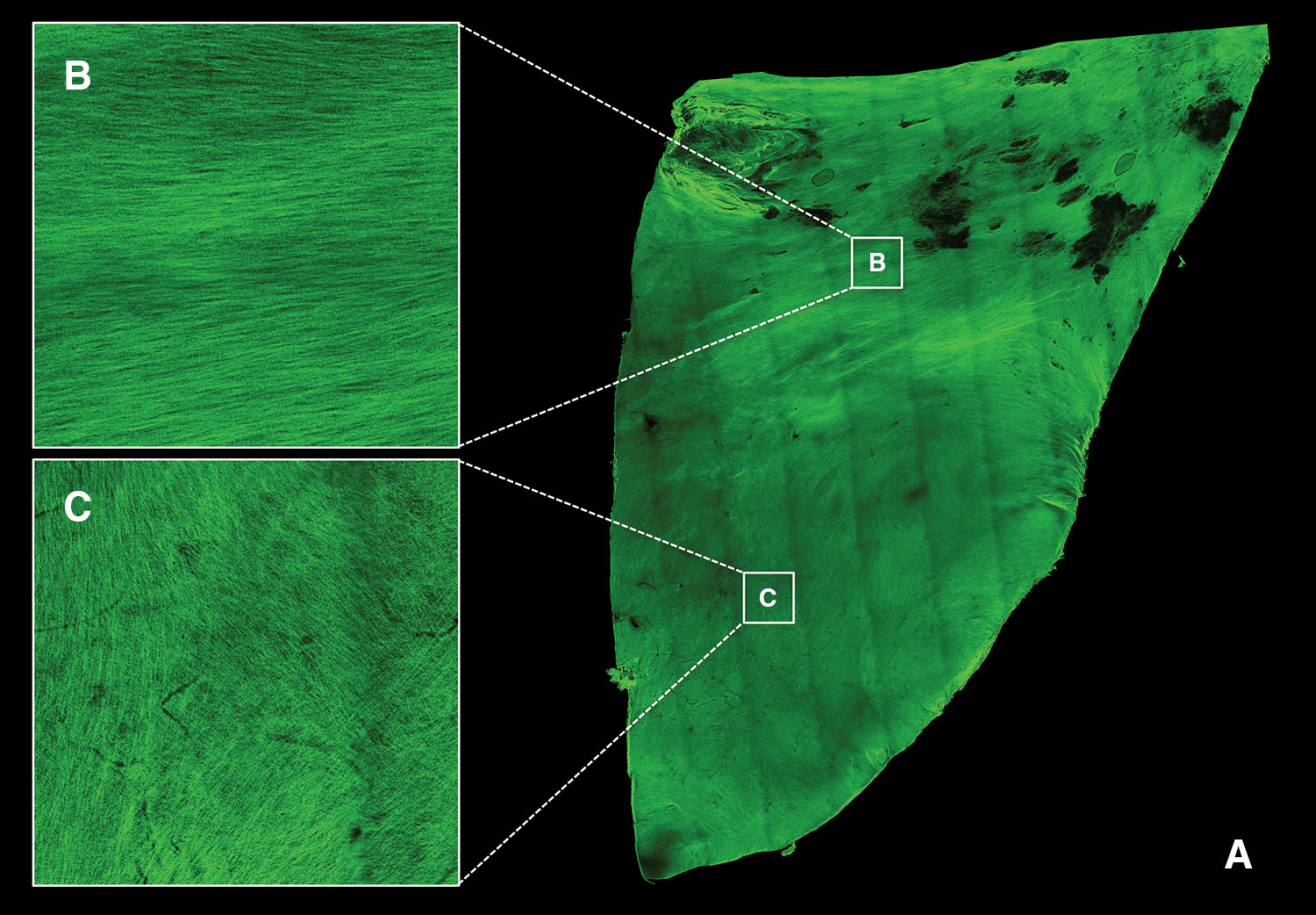
Global collagen orientation. Representative image of a collagen specific whole mount staining on half a DTEHV leaflet, visualized by confocal microscopy at the ventricular side (a), revealed that collagen orientation was clearly aligned in circumferential direction in the coaptation area (b), and randomly distributed towards the bottom of the belly (c). Black spots indicate local regions that were out of focal plane.

By analyzing the collagen whole mount stainings, global collagen orientation was visualized throughout ~200 μm in depth, over the entire half of 5 DTEHV leaflets. A representative maximum projection in *Z*-direction is shown in Fig. 5a. Here, the collagen was clearly aligned in circumferential direction in the coaptation area (Fig. 5b) and more randomly distributed towards the bottom region of the belly (Fig. 5c).

### Tissue Mechanics and *In Vivo* Collagen Remodeling Simulations

#### Biomechanical Properties

**Figure 6:**
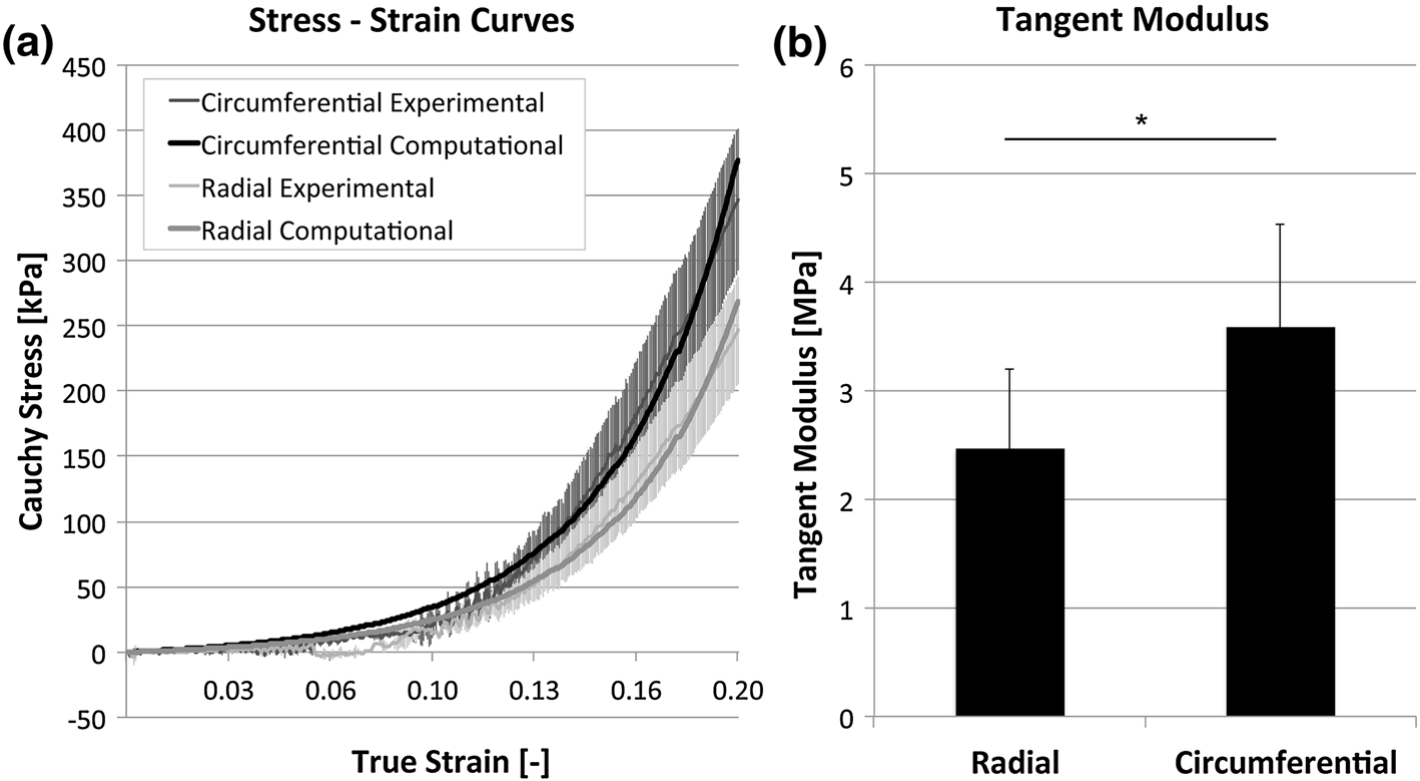
Mechanical properties of the DTEHVs. The averaged stress–strain curves obtained from equal biaxial tensile tests of the control group, in both radial and circumferential direction, shows Cauchy stresses ranging between the 200–400 kPa (a). The fitted curves are used as material parameters for the computational simulations (a). The calculated tangent moduli of all DTEHVs showed a significant increase in tissue stiffness in circumferential direction compared to the radial direction (*p = 0.035) (b). All values are represented as mean values ± standard deviation.

The averaged tensile curves from the control valve showed Cauchy stresses ranging between 200–300 kPa in radial direction and 300–400 kPa in circumferential direction at 20% strain (Fig. 6a), indicative for tissue anisotropy. From the averaged equibiaxial tensile tests on all valves, it appeared that the tangent moduli were significantly higher in circumferential direction, compared to the radial direction (*p* = 0.035), being 3.59 ± 0.95 and 2.47 ± 0.74 MPa respectively at 20% strain (Fig. 6b).

#### Computational Simulations

Fitting the numerical model on the experimental tensile data of the control group (Fig. 6b), resulted in the following parameter values: $k_{1} = 2 2. 0\; {\text{kPa}}, \;k_{2} = 7. 5 0, \;{\text{and}} \;\sigma = 6 7. 5^{^\circ } .$

**Figure 7:**
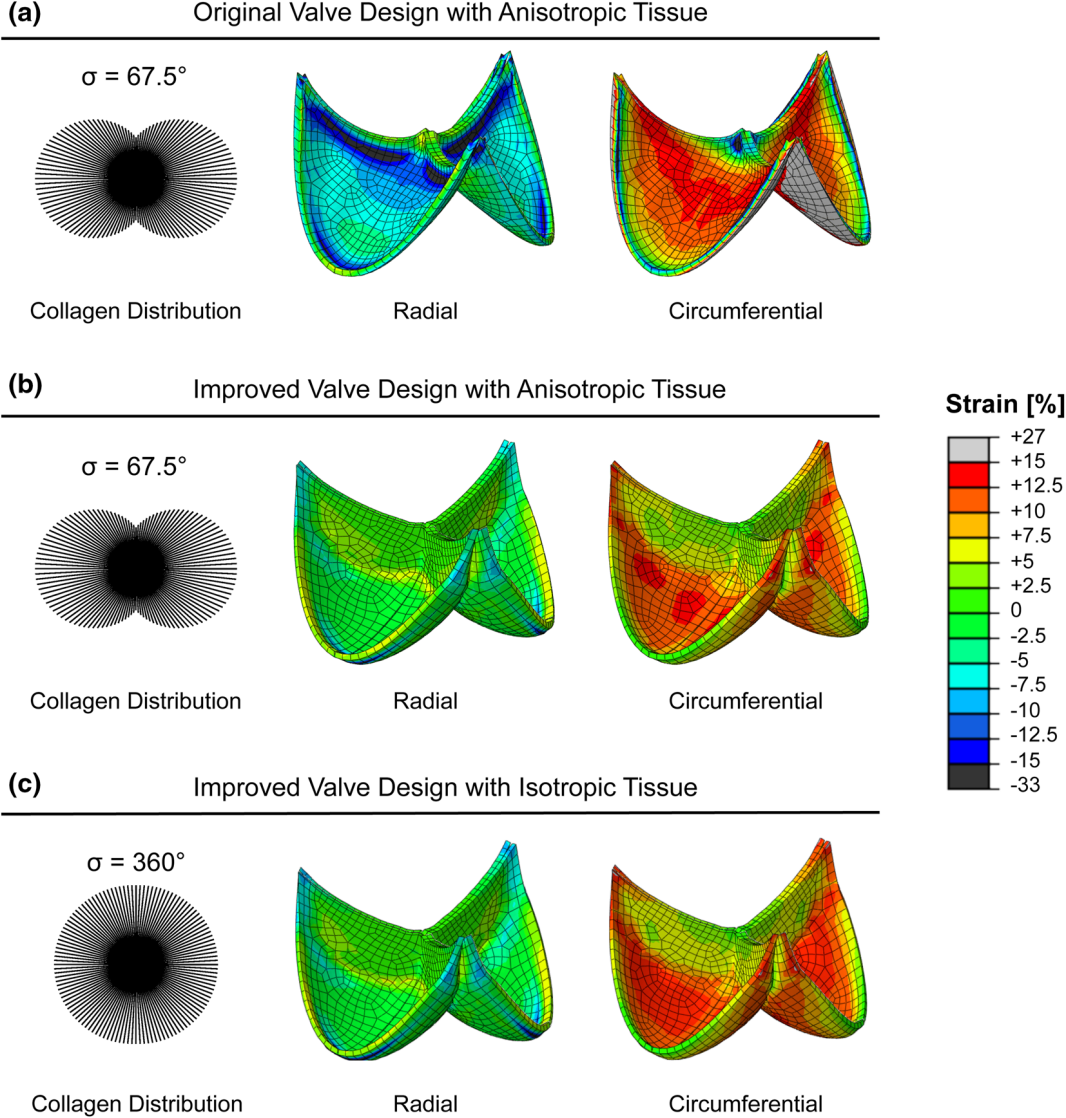
Computational simulations. Material parameters of the DTEHVs were implemented into an established computational valve model, to assess the strains in radial and circumferential direction in both the original and the improved valve design, simulating 15 mmHg mean pulmonary pressure. In the original valve design the entire leaflet tissue is being compressed in radial direction (a). The improved valve design showed a considerable decrease in radial compression in the belly (b), which is primarily due to geometrical improvements rather than collagen anisotropy (c). Strains in circumferential direction are comparable between both valve designs.

At a mean pulmonary differential pressure of 15 mmHg the computational simulations revealed that the original design was subjected to leaflet tissue compression in the radial direction throughout the entire leaflet during loading (Fig. 7a). The improved valve design of these DTEHVs showed a considerable decrease in radial tissue compression as compared to the original design (Fig. 7b). Strains in the circumferential direction were maintained and comparable in both designs. Besides, the effect of the present collagen anisotropy on tissue loading in the improved design, revealed no changes in radial tissue compression compared to fully isotropic collagen orientation (Fig. 7c).

## Discussion

From our first long-term *in vivo* experiments where we implanted DTEHVs in sheep, we observed leaflet fusion with the wall, which resulted in the development of valvular insufficiency over time.^6^,^30^ Based on computational simulations it was hypothesized that the original DTEHV geometry had to be adjusted to enable leaflet extension in the radial direction, rather than compression. Therefore, a bioreactor insert was developed to impose the desired valvular geometry during culture, which was maintained after decellularization and removal of the insert. This resulted in DTEHVs with an increased coaptation area and a significant radial and circumferential belly curvature.

Adjusting the culture conditions in the bioreactor system by introducing a constraining insert might have affected tissue formation by limiting nutrient and oxygen exchange. However, it appeared that these DTEHVs still contain a uniform ECM distribution throughout the entire thickness of the leaflets, consisting mainly of collagen. These histological findings are in agreement with previously cultured human cell-based TEHVs having the original valve geometry without the insert.^16^

Further more the global collagen orientation was influenced by introducing the insert. During tissue culture, non-woven PGA meshes are known to hydrolyze, thereby losing their mechanical integrity and structural support.^9^,^19^,^23^ This degradation profile in combination with the tension forces exerted by the cells, led to tissue compaction in the unconstrained directions, which resulted in collagen orientation along the constrained direction.^4^,^18^

In this study, tissue compaction against a rigid object was used to impose the DTEHV geometry according to the shape of the bioreactor insert. After 2 weeks of culture, the scaffold loses its mechanical integrity and the tissue starts to compact.^29^ While compacting, the leaflet tissue is being constrained by the rigid bioreactor insert, except for the tissue at the free edges of the leaflets that could still compact slightly in the radial direction. This resulted in mainly circumferential aligned collagen in the coaptation area, and a more random distribution towards the bottom of the belly.

Other studies in which constraining methods were used to control the geometry of DTEHVs are promising, however no long-term functionality up to 12 million cycles have been reported so far.^18^,^24^–^26^,^28^ The DTEHVs created in the present study showed satisfactory long-term functionality up to 16 weeks *in vivo* simulation in terms of regurgitation, cardiac output and opening and closing behavior, with only one valve failing after 4 million cycles, being unable to adapt and stabilize to the applied pulmonary pressure conditions during fatigue testing. From previous *in vivo* implantation studies in both sheep and non-human primates, host cell repopulation was observed within 5 h, accompanied by changes in the extracellular matrix after 8 weeks, with evidence of ECM production by these cells.^6^,^30^ Therefore, the DTEHVs as developed in this study are expected to be sufficiently fatigue resistant under physiological pulmonary conditions, to provide sufficient time for host cells to repopulate and maintain the ECM.

In addition to the improved valvular geometry in terms of a large coaptation area and an enhanced belly curvature, collagen anisotropy is essential to obtain radial leaflet stretch during dynamic loading, characteristic for native leaflets,^2^ were anisotropy is expected to further increase after *in vivo* implantation because of strain-induced collagen reorganization by the repopulating host cells.^4^ Compared to reported stiffness values,^1^ human native aortic heart valve leaflets have a tangent modulus in radial and circumferential direction of about 2.0 ± 1.5 and 15.6 ± 6.4 MPa respectively. The reported tangent modulus of the DTEHVs in radial direction is comparable with 2.5 ± 0.7 MPa, but is lower in circumferential direction with 3.6 ± 1.0 MPa.

Despite that local observed differences in collagen anisotropy were not implemented into the model, these computational simulations revealed that the additional effect of the overall collagen anisotropy seems not to influence the tissue loading behavior under pulmonary loading conditions. Therefore the implemented geometrical improvements only, were already sufficient to prevent radial tissue compression almost completely in the entire valve.

The concept of using DTEHVs for human applications still holds great promise in terms of regenerative capacity and growth potential, which would overcome the necessity for multiple re-interventions in young patients. The use of autologous cells is not required and allogeneic cells can be used that simplifies regulations and allows for off-the-shelf availability. Further development on biodegradable stents that are suitable for minimal invasive heart valve implantation should be focus of future studies to complete the growing valve concept.

In conclusion, this study proposes a successful solution to impose a desired three-dimensional curved tissue engineered valvular geometry by using a constraining bioreactor insert during culture, which allows for a maintained shape after decellularization and removal of the insert. This resulted in fully competent off-the-shelf available human cell-based DTEHVs with a large coaptation area and profound belly curvature. Long-term functionality was maintained mainly up to 16 weeks *in vivo* simulation, allowing sufficient time for host cell repopulation. Usage of the bioreactor inserts resulted in homogeneously distributed tissue formation, circumferential collagen orientation in the coaptation region, and overall leaflet tissue anisotropy. Based on the mechanical data, our computational models revealed a considerable decrease in radial tissue compression with the obtained geometrical adjustments. Therefore, these improved DTEHV are expected to be less prone to host cell mediated leaflet retraction and will remain competent after implantation.
